# Uncertain Associations of Major Bleeding and Concurrent Use of Antiplatelet Agents and Chinese Medications: A Nested Case-Crossover Study

**DOI:** 10.1155/2017/9417186

**Published:** 2017-08-02

**Authors:** Hsin-Hui Tsai, Hsiang-Wen Lin, Chiu-Lin Tsai, Felix K. Yam, Sheng-Shing Lin

**Affiliations:** ^1^Department of Cosmetic Science, Providence University, 200, Sec. 7, Taiwan Boulevard, Taichung 43301, Taiwan; ^2^Department of Nursing, National Taichung University of Science and Technology, 193, Sec. 1, Sanmin Road, Taichung 40343, Taiwan; ^3^Department of Pharmacy, Yuan Rung General Hospital, 201 Zhongzheng Road, Yuanlin City, Changhua 510, Taiwan; ^4^School of Pharmacy and Graduate Institute, College of Pharmacy, China Medical University, 91 Hsueh-Shih Road, Taichung 40402, Taiwan; ^5^Department of Pharmacy, China Medical University Hospital, 2 Xue Shi Road, Taichung 40402, Taiwan; ^6^UC San Diego Skaggs School of Pharmacy and Pharmaceutical Sciences, 9500 Gilman Drive, La Jolla, CA 92093, USA; ^7^Department of Chinese Medicine, China Medical University Hospital, 2 Xue Shi Road, Taichung 40402, Taiwan; ^8^Graduate Institute of Chinese Medicine, College of Chinese Medicine, China Medical University, 91 Hsueh-Shih Road, Taichung 40402, Taiwan

## Abstract

Despite the evidence that some commonly used Chinese medications (CMs) have antiplatelet/anticoagulant effects, many patients still used antiplatelets combined with CMs. We conducted a nested case-crossover study to examine the associations between the concomitant use of antiplatelets and CMs and major bleeding using population-based health database in Taiwan. Among the cohort of 79,463 outpatients prescribed antiplatelets (e.g., aspirin and clopidogrel) continuously, 1,209 patients hospitalized with new occurring bleeding in 2012 and 2013 were included. Those recruited patients served as their own controls to compare different times of exposure to prespecified CMs (e.g., Asian ginseng and dong quai) and antiplatelet agents. The periods of case, control 1, and control 2 were defined as 1–4 weeks, 6–9 weeks, and 13–16 weeks before hospitalization, respectively. Conditional logistic regression analyses found that concurrent use of antiplatelet drugs with any of the prespecified CMs in the case period might not significantly increase the risks of bleeding over that in the control periods (OR = 1.00, 95% CI 0.51 to 1.95 and OR = 1.13, 95% CI 0.65 to 1.97). The study showed no strong relationships between hospitalization for major bleeding events and concurrent use of antiplatelet drugs with the prespecified CMs.

## 1. Introduction

The use of aspirin and/or other antiplatelet therapies for adults with or at risk of cardiovascular disease (CVD) is strongly recommended by international guidelines [1, 2]. The risks of bleeding do not outweigh the benefits of antiplatelet therapy [3, 4]. Advanced age, female gender, multiple comorbidities, and concomitant use of antiplatelet and anticoagulant drugs were found to be risk factors for bleedings [5, 6]. While 2 to 46% of patients with CVD ever took herbal products [7, 8], 20% of CVD patients had a previous history of concomitant use of antiplatelet agents with herbal medicines [9].

Despite a lack of scientific evidence to support the use of Traditional Chinese Medicine (TCM), TCM is still common in Asian countries and is also increasing in popularity in Western countries. More than 60% of the Taiwan's National Health Insurance (NHI) beneficiaries have utilized TCM services, and approximately 86% of TCM visits resulted in prescriptions for Chinese medications (CMs) [10]. Despite studies showing that commonly used CMs (e.g., danshen, dong quai, and licorice) may have antiplatelet/anticoagulant effects [11, 12], many patients still used antiplatelet agents combined with CMs [9, 13]. Our previous study showed that the prevalence of concurrent use of antiplatelet agents with CMs was 13%, as the average duration of using antiplatelets with CMs was about 26 days in one year [14]. It implied that the concurrent use of CMs is usually intermittent rather than continuous. The aim of this study was to examine the transient events of concurrent use of antiplatelet drugs with a set of prespecified CMs and its association with hospitalization due to major bleeding events.

## 2. Materials and Methods

### 2.1. Data Source

The NHI in Taiwan was launched in 1995 and has enrolled more than 99% of the Taiwanese population. The majorities of marketed Western medications are evaluated by the Taiwan Food and Drug Administration and are covered by the NHI Program. Powdered, concentrated CM products, which are manufactured by pharmaceutical factories that comply with the current good manufacture practice, are almost covered. We utilized the 2000 and 2005 Longitudinal National Health Insurance Databases, from which two million beneficiaries were randomly selected from all of the registered beneficiaries (e.g., 23.28 million persons in 2012). The variables of interest to evaluate the outcomes and associations included patients' disease statuses and medication use during the study periods. Our study was exempt from the Institutional Review Board because the NHI research database contains the deidentified person identifiers and has been made publicly available through a rigorous application process.

### 2.2. Study Design

Upon the literature review, it seems more popular to use case-crossover study design to evaluate drug safety concerns with transient exposure and corresponding abrupt outcomes in the area of pharmacoepidemiology [15, 16]. In this study, the outcome of interest was hospitalization due to major bleeding events, which were considered as acute and transient events among survivors. Thus, we first identified a cohort of antiplatelet users and then performed the case-crossover study design to examine the relationships between the major bleeding events and concomitant use of antiplatelet agents with CMs. In contrast to a case-control or cohort study, those who were ever hospitalized for major bleeding would serve as his or her own control to compare different times of exposure, rather than comparing the same time period across different individuals. In this case, such study design is expected to effectively decrease the selection bias and reduce the influence of confounders related to individuals in those studies regarding drug interactions [17, 18].

### 2.3. Study Population

Patients who were prescribed with antiplatelet agents (aspirin, clopidogrel, dipyridamole, or ticlopidine) continuously for at least 180 days in outpatient settings between January 2012 and December 2013 were included in this open cohort. If there was more than one exposure to antiplatelet agents during the observation period, only the first exposure was considered to calculate its continuous use for each patient. Those antiplatelet agents that were approved to be used in Taiwan after 2012 (i.e., ticagrelor) and administered as an injection (e.g., tirofiban, eptifibatide, and abciximab) were not included. Enrolled patients were followed from the day of first antiplatelet prescription to the day of hospitalization due to major bleeding, discontinuation of antiplatelet agents, or the end of the study (31 December 2013), whichever came first. Major bleeding was defined as a diagnosis of gastrointestinal (GI) bleeding, intracranial bleeding, nose/eye bleeding, urogenital bleeding, and others. If the number of days between the last date of an antiplatelet prescription and the start date of the following antiplatelet prescription exceeded seven days, it was defined as discontinuous use. The date of hospitalization due to major bleeding was identified as the index date. For each patient, only the first hospitalization during the observation period was considered. Furthermore, we excluded those who had a history of major bleeding within the six months prior to the first exposure to antiplatelet agents as cases for further analysis in this study.

### 2.4. Exposure to Chinese Medications

The following concentrated, NHI covered CMs, which have been reported to have evidence of bleeding risks [12], were identified as the “prespecified” CMs: American ginseng* (Panax quinquefolius)*, Asian ginseng* (Panax ginseng)*, danshen* (Salvia miltiorrhiza)*, dong quai* (Angelica sinensis)*, garlic* (Allium sativum)*, ginger* (Zingiber officinale)*, licorice* (Glycyrrhiza glabra)*, Siberian ginseng* (Eleutherococcus senticosus)*, and turmeric* (Curcuma longa)*. We further evaluated their risks of bleeding with antiplatelets. The use of any single CM or CM formula (i.e., 244 items of CM formula in total) with these prespecified CMs during the study period was defined as an exposure, which could be the source of potential interactions with antiplatelets.

Previous studies have shown that the majority of bleeding events in patients using dual antiplatelet therapy occurred in the 2–4 weeks following the initiation of the combination [4, 19]. Applying the same concept, we hypothesized that the concurrent use of antiplatelet agents and prespecified CMs would increase the risks of bleeding over a similar time frame. Thus, we defined the case period as 1–4 weeks prior to the index date (i.e., hospitalization due to major bleeding), while control period 1 and control period 2 were identified as 6–9 weeks and 13–16 weeks prior to the index date, respectively. The detailed exposure patterns of prespecified CMs before hospitalization due to major hemorrhage events among antiplatelet users during each study period were further examined. In a sensitivity analysis, we performed the same analysis approach on a different cohort of antiplatelet users (those who were NHI beneficiaries in 2010-2011) to compare the effects of the prespecified CM use between case period and control periods.

### 2.5. Potential Confounders

We controlled for comorbidities and comedications during these study periods, which might contribute to an increase or reduction of bleeding risks among antiplatelet users. The following comorbidities were included: cancer, cerebrovascular accidents, coronary artery diseases, diabetes, heart failure, hypertension, liver disease, obesity, and renal failure. Medications that potentiate or decrease the risks of bleeding were controlled for further analysis. The included medications were as follows: (1) glucocorticoids, nonsteroidal anti-inflammatory drugs, selective serotonin reuptake inhibitors, statins, and warfarin; and (2) selective histamine type 2 receptor antagonists (H2 blockers), proton-pump inhibitors, and cytoprotective agents (e.g., misoprostol and sucralfate) [17, 20–22].

### 2.6. Statistical Analyses

During the observation period, we calculated the period prevalence and incidence of hospitalization related to major bleeding occurrences among antiplatelet users. The number of patients hospitalized due to major bleeding was the numerator and all antiplatelet users included in the cohort were the denominator in the estimation of the period prevalence. Instead, the number of patients hospitalized due to major bleeding minus the number of patients with the prior history of bleeding was used as numerator to calculate the incidence.* Chi*-square tests were used to compare differences in comorbidities and comedications during case and control periods. Conditional logistic regression analysis was performed to determine the associations between major bleeding and exposure to concomitant use of the prespecified CMs and antiplatelet agents. A univariate regression model was used to estimate the crude odds ratios (ORs), while a multivariate regression model was used for adjusted ORs. All the listed “potential confounders” in the prior section were included in the multivariate model. A *p* value < 0.05 was considered statistically significant. The ORs and 95% confidence intervals (CIs) were calculated for overall use of any CMs and each individual CM. All analyses were conducted using SAS version 9.4.

## 3. Results

**Figure 1:**
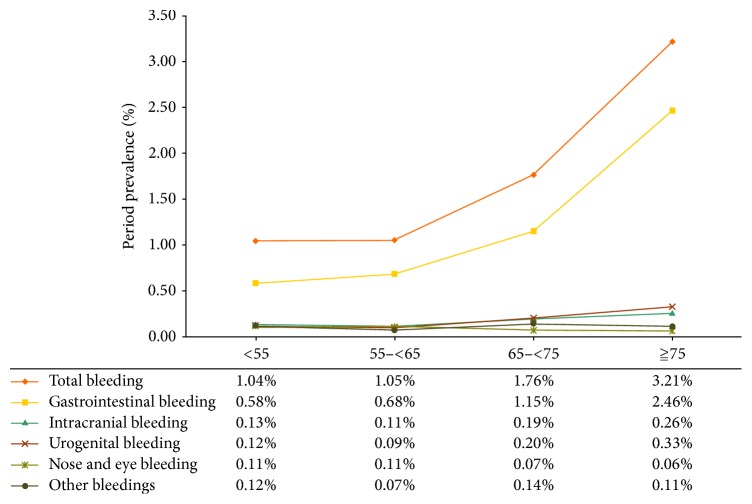
The period prevalence of major bleeding by age group among antiplatelet users.

During the study period of 2012-2013, 79,463 continuous antiplatelet users were identified and were followed for 86,615 person-years. The average follow-up time was 397.5 ± 177.0 days. Of them, 1,516 patients (1.91%) were identified to have had hospitalization due to major bleeding events. The majority of those patients (*n* = 1,068, 70.45%) were hospitalized for GI bleeding, followed by urogenital bleeding (*n* = 158, 10.42%), intracranial bleeding (*n* = 145, 9.56%), and nose/eye bleeding (*n* = 67, 4.42%) (Table 1). The period prevalence of total bleeding events and GI bleeding events among antiplatelet users increased with age (Figure 1), especially in the elderly (i.e., greater than 65 years old). 

**Figure 2:**
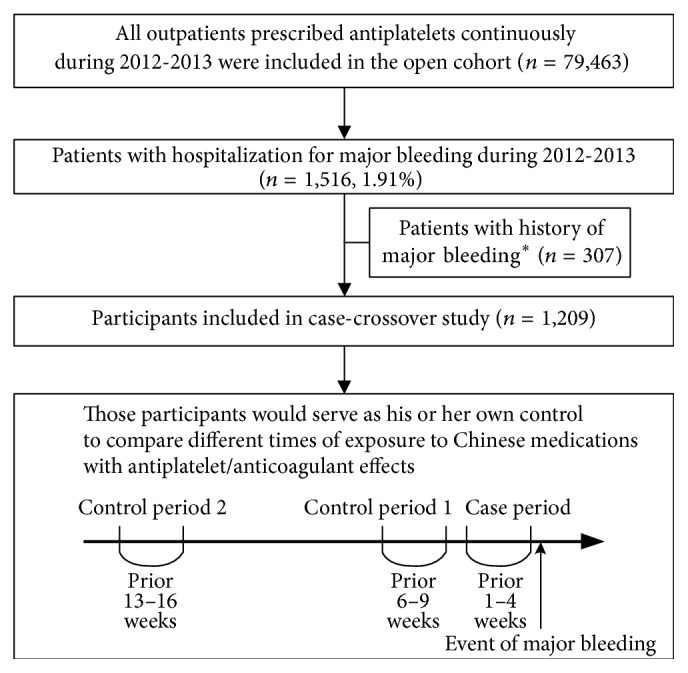
*Flow chart of participants included in the study*. ^*∗*^Patients had a history of major bleeding within the six months prior to the first date of prescription of antiplatelet agents during 2012-2013.

After excluding patients with a history of major bleeding, 1,209 patients were included for further analysis (Figure 2). More than half of the antiplatelet users with incidents of major bleeding were male (*n* = 742, 61.4%). The mean age of included participants was 74.1 ± 11.7. The incidence of any major bleeding was 1.52% (14.0 per 1000 person-years).  Table 2 shows that more patients had a diagnosis of hypertension and renal failure during the case period (1–4 weeks prior to the index date) when compared with control period 2 (13–16 weeks prior to the index date). In addition, more patients had a prescription for nonsteroidal anti-inflammatory drugs or a prescription for H2 receptor antagonists and proton-pump inhibitors during the case period (1–4 weeks prior to the index date) when compared with the both control periods (6–9 weeks or 13–16 weeks prior to the index date).

No prescription records for American ginseng, garlic, or Siberian ginseng were found in the TCM outpatient records of the included NHI beneficiaries in the study periods. The detailed exposure patterns of prespecified CMs before hospitalization due to major hemorrhage events during each study period were described in Appendix  1 in Supplementary Material available online at https://doi.org/10.1155/2017/9417186. In total, there were 97 antiplatelet users ever exposed to specified CM during case and/or control periods before hospitalization due to major bleedings (Appendix  1 in Supplementary Material). Of them, 62, 60, and 56 antiplatelet users had ever been prescribed with the specified CMs during case, control 1, and control 2 periods, respectively (Table 3). Specifically, 42 and 32 among 62 antiplatelet users were prescribed with those CMs in both case/control period 1 and case/control period 2, respectively (Table 3). Twenty-nine patients were prescribed CMs exactly one day before the hospitalization due to major bleedings (with the exposure days ranging from 1 day to more than 2 weeks) and 18 patients were prescribed specified CMs more than 14 days during the closest two-week periods prior to hospitalization due to major hemorrhage events. Some other patients were prescribed CMs only in either control period 1 and/or control period 2 but not in case period (Appendix  1 in Supplementary Material).

To compare with control periods 1 and 2, the adjusted ORs (with a 95% CI) for risk of bleeding events were 1.00 (0.51–1.95) and 1.13 (0.65–1.97), respectively (Table 3). Most of those patients were prescribed with the CM formula; thus the ORs of any of prespecified CM formula were similar to the results of any use of prespecified CM. Although the OR of any of prespecified single CM was less than 1, the CI showed that there was no significant difference. Concurrent use of antiplatelet drugs with Asian ginseng in the case period increased the risk of bleeding more than that in the control period 2. The adjusted ORs were 3.27 (1.09–9.83). The use of danshen, dong quai, and ginger seemed to decrease the risk of bleeding, while these results did not reach statistical significance. The ORs of concurrent use of antiplatelets with licorice were similar to the results of any use of prespecified CM (Table 3). Some of the statistically significant differences and directions of outcomes varied in the sensitivity analysis. For instance, all of the adjusted ORs for antiplatelet users ever exposed to any of the individual CM (including Asian ginseng) were not statistically significant in the sensitivity analysis in 2010-2011 (Appendix  2 in Supplementary Material).

## 4. Discussion

We found that the period prevalence and incidence of any major bleeding events were 1.91% and 1.52%, respectively, among antiplatelet users. More than 70% of the bleeding events were attributed to GI bleeding. The use of any prespecified CMs (i.e., Asian ginseng, danshen, dong quai, ginger, licorice, and turmeric) in the cohort might not increase the risk of major bleeding event significantly. The relationship between hospitalization for major bleeding and concurrent use of antiplatelet agents with any individual CM was not robust.

The low prevalence of hospitalization due to the major bleedings (i.e., 1.91%) among the antiplatelet users in Taiwan was similar to the findings obtained in a meta-analysis [23]. The rates of major bleeding for aspirin, dipyridamole, clopidogrel, and ticlopidine were 1.0–2.5%. Moreover, the prevalence of major bleeding, especially GI bleeding, among antiplatelet users increased as the patients' ages increased. This finding is consistent with other review articles [5]. Polypharmacy and polyherbacy are common among the elderly population and may be associated with a greater propensity for drug interactions [24–26].

There is conflicting evidence on the potential effects of Asian ginseng with antiplatelet and anticoagulant agents. An in vitro study found that a crude extract of Asian ginseng and its active component, ginsenoside Rg2, had strong anticoagulant effects [27]. However, the concurrent use of warfarin and Asian ginseng had no effect on international normalized ratios in a randomized crossover study [28]. Furthermore, the in vitro and in vivo data have demonstrated that “unprocessed” Asian ginseng has fewer antiplatelet effects compared to “processed” Asian ginseng [29]. Although we did not observe a robust association between concomitant use of antiplatelet agents and Asian ginseng in the occurrence of major bleeding events in this study, the significant associations between case period and farther period (i.e., control period 2), regardless of controlling for other factors, were observed but these might have occurred due to chance. Nevertheless, it is still recommended that healthcare professionals still should be aware of the possible effects of Asian ginseng on antiplatelet and anticoagulant agents.

The integration of conventional medicine with complementary and alternative medicine, including TCM (e.g., using Asian ginseng as medications or in herbal cuisine), is becoming popular all over the world [30, 31]. In particular, the combination of Western medication and CM is increasingly prevalent and commonly used to treat many chronic diseases in the Chinese population, including CVD [32–36]. For instance, a meta-analysis demonstrated that the addition of CM to conventional therapy for patients with myocardial infarction could reduce the mortality but could also increase the risk of bleeding [34].

We did not identify a strong relationship between major bleeding events and concomitant exposure to antiplatelet agents and any individual prespecified CMs in this study. The impact of the selected CMs on bleeding risks seemed various across different individual CMs. We assumed those CMs with multiple ingredients, with different concentrations of the active ingredients, and various processing procedures may be the reasons for the inconsistent findings [37]. Furthermore, the composition of a CM formula is usually based on the common, conventional principle of a drug remedy, that is, prescribing based on the roles of drugs as monarch, minister, adjuvant, or guide. Those CMs recognized as “monarch” drugs usually have major amounts of the ingredients and play important roles. In contrast, CMs categorized as guiding drugs are medications with relatively minor amounts to regulate the properties of the other CMs [38]. These guiding CMs are frequently used. For instance, licorice is the most common guiding drug and is contained in more than half of CM formulas to increase the effectiveness, to reduce the toxicity, or to improve the flavor of the other ingredients [39]. The majority of antiplatelet users who used CMs were also exposed to licorice in this study so that the crude ORs of licorice were similar to the results obtained from any use CMs.

Several limitations were unavoidable in this study and need to be addressed. Firstly, we only included antiplatelet users who had encountered the observed outcomes of hospitalization due to major bleeding events. Patients who suffered from mild or moderate bleeding but do not require to be hospitalized (i.e., visiting in outpatient clinics or the emergency department) and could die due to massive bleeding were not evaluated in this study. Secondly, only NHI covered concentrated CMs were included so that the clinical consequences of decoction pieces of crude CMs or of dietary supplement products with antiplatelet agents were not evaluated. Thirdly, this study did not include some other additional factors which might be associated with major bleeding. Dual antiplatelet therapy or combination with the new oral anticoagulants was not evaluated. The dosing of the antiplatelet medications or CMs was not considered in this study. The study used a predefined list of CMs with evidence for antiplatelet or anticoagulant effect and might miss those additional CMs or CM formulas which also have anticoagulant or antiplatelet properties. Moreover, the lifestyle data (i.e., smoking and drinking history and body mass index) was not evaluated due to lack of data in the study database. Adherence to prescribed medications and CMs was also not able to be assessed using the NHI database. In addition, only the first three and five diagnoses were included in outpatient and inpatient databases of NHI databases, respectively. Thus, only the most important diagnoses (i.e., the most severe diseases) could be identified during each of the study periods. As a result, we performed a case-crossover study instead of a conventional case-control or cohort study to reduce the influence of these confounders. Further, the length of exposure windows for case and control periods prior to the hospitalization due to major bleedings (e.g., 1–4 weeks versus 13–16 weeks) might be the other contributing factor for the various associations as well. Lastly, a case-crossover study is typically performed when events are considered acute and transient. We made the assumption that bleeding events leading to hospitalization were acute events triggered by a recent occurrence. This method may underestimate the bleeding risk that is present throughout treatment. However, a similar case-crossover study approach was used in the other studies evaluating the risk of acute myocardial infarction based on the time of day [40, 41].

## 5. Conclusions

The findings of this population-based study showed that the risk of major bleeding associated with combination of any prespecified CMs (including Asian ginseng, danshen, dong quai, ginger, or licorice) with antiplatelet agents was not ascertained. Regardless, healthcare professionals still need to carefully monitor and assess bleeding risks on those patients who are taking CMs and are prescribed antiplatelet agents. Further studies are needed to evaluate the benefits of concomitant use of antiplatelet agents and CMs in the integrative therapy in the future.

## Supplementary Material

Appendix 1: The detailed use pattern of any of the prespecific Chinese medications during 2012-2013.
Appendix 2: Results of sensitive analyses using the cohort with the same criteria and same case and control periods of 2010-2011 databases.

## Figures and Tables

**Table 1:** The period prevalence and incidence of major bleeding among antiplatelet users.

	Period prevalence		Incidence		
	Patient number	%^a^	Patient number	%^a^	Per 1000 person-years^b^
Ever encountered bleeding events	1516	1.91%	1209	1.52%	14.0
Gastrointestinal bleeding	1068	1.34%	865	1.09%	10.0
Urogenital bleeding	158	0.20%	121	0.15%	1.4
Intracranial bleeding	145	0.18%	113	0.14%	1.3
Nose and eye bleeding	67	0.08%	39	0.05%	0.5
Other bleedings	85	0.11%	75	0.09%	0.9

^a^The total number of patients included in the cohort was 79,463; ^b^the total number of person-years was 86,615.

**Table 2:** Comparisons of comorbidities and concomitant medications among antiplatelet users between case period and control periods.

	Case period^a^	Control period 1^b^	*p* value	Control period 2^c^	*p* value^d^
*Comorbidity*					
Hypertension	423 (35.0%)	395 (32.7%)	0.2288	370 (30.6%)	0.0217^*∗*^
Coronary artery diseases	250 (20.7%)	232 (19.2%)	0.3595	222 (18.4%)	0.1508
Renal failure	146 (12.1%)	133 (11.0%)	0.4080	114 (9.4%)	0.0357^*∗*^
Diabetes	108 (8.9%)	119 (9.8%)	0.4431	113 (9.4%)	0.7242
Heart failure	79 (6.5%)	65 (5.4%)	0.2290	61 (5.1%)	0.1170
Cerebrovascular accident	57 (4.7%)	48 (4.0%)	0.3692	48 (4.0%)	0.3692
Cancer	51 (4.2%)	43 (3.6%)	0.4000	35 (4.2%)	0.0789
Liver disease	44 (3.6%)	28 (2.3%)	0.0556	39 (3.2%)	0.5765
Obesity	1 (0.1%)	1 (0.1%)	1	1 (0.1%)	1
*Concomitant medication*					
Increasing risk of bleeding					
NSAIDs	1028 (85.0%)	968 (80.1%)	0.0013^*∗*^	932 (77.1%)	<0.0001^*∗*^
Statins	291 (24.1%)	281 (23.2%)	0.6323	269 (22.3%)	0.2889
Glucocorticoids	97 (8.0%)	81 (6.7%)	0.2128	76 (6.3%)	0.0975
SSRI	37 (3.1%)	36 (3.0%)	0.9054	32 (2.7%)	0.5414
Warfarin	25 (2.1%)	22 (1.8%)	0.6586	23 (1.9%)	0.7706
Prevent risk of bleeding					
H2 blockers	220 (18.2%)	178 (14.7%)	0.0213^*∗*^	165 (13.7%)	0.0022^*∗*^
PPI	64 (5.3%)	44 (3.6%)	0.0490^*∗*^	42 (3.47%)	0.0289^*∗*^
Cytoprotective agents	4 (0.3%)	0	0.0453^*∗*^	6 (0.5%)	0.5262

NSAID: nonsteroidal anti-inflammatory drugs; SSRI: selective serotonin reuptake inhibitors; PPI: proton-pump inhibitors; ^a^1–4 weeks prior to the index date; ^b^6–9 weeks prior to the index date; ^c^13–16 weeks prior to the index date; ^d^significance is reached when ^*∗*^*p* < 0.05.

**Table 3:** Potential risk of major bleeding associated with concurrent use of prespecified Chinese medications with antiplatelet agents^a^.

CM^b^	Number of users in different periods (Case versus control period 1)			Exposure odds ratio (95% confidence interval)		Number of users in different periods (case versus control period 2)		Exposure odds ratio (95% confidence interval)	
	Case period^c^	Control period 1^d^	Both periods 1^e^	Crude	Adjusted	Control period 2^f^	Both periods 2^g^	Crude	Adjusted
Any of prespecified CM^h^	62	60	42	1.11 (0.59–2.10)	1.00 (0.51–1.95)^i^	56	32	1.23 (0.73–2.07)	1.13 (0.65–1.97)^j^
Any of prespecified single CM^k^	16	15	6	1.11 (0.45–2.73)	0.95 (0.38–2.40)^i^	16	8	1.00 (0.38–2.66)	0.76 (0.27–2.15)^j^
Any of prespecified CM formula^l^	61	60	41	1.05 (0.56–1.97)	0.94 (0.48–1.81)^i^	55	29	1.23 (0.73–2.07)	1.12 (0.64–1.94)^j^
Licorice^m^	62	58	42	1.25 (0.65–2.41)	1.19 (0.28–5.09)^n^	53	29	1.38 (0.81–2.33)	1.10 (0.35–3.42)^o^
Ginger^m^	49	45	30	1.27 (0.64–2.49)	0.84 (0.19–3.73)^n^	40	18	1.41 (0.82–2.43)	0.72 (0.25–2.08)^o^
Asian ginseng^m^	41	37	24	1.31 (0.64–2.70)	1.49 (0.38–5.88)^n^	26	13	2.15 (1.12–4.16)_ _^*∗*^	3.27 (1.09–9.83)_ _^*∗*o^
Dong quai^m^	39	39	23	1.00 (0.50–2.00)	0.95 (0.29–3.08)^n^	35	16	1.21 (0.66–2.22)	0.86 (0.29–2.58)^o^
Danshen^m^	7	9	4	0.60 (0.14–2.51)	0.37 (0.08–1.78)^n^	11	5	0.33 (0.07–1.65)	0.17 (0.02–1.16)^o^
Turmeric^m^	1	0	1	—	—	1	0	1.00 (0.06–15.99)	0.52 (0.02–17.50)^o^

CM: Chinese medication; OR: odds ratio; CI: confidence intervals. ^a^The total number of participants included in case-crossover study was 1,209; ^b^there were no patients prescribed American ginseng, garlic, and Siberian ginseng during the case or control period; ^c^the number of patients exposed to the prespecified CMs during 1–4 weeks prior to the index date; ^d^the number of patients exposed to the prespecified CMs during 6–9 weeks prior to the index date; ^e^the counts of concordant users were subtracted from total counts in case and control period 1 to give the numbers of discordant users for calculating the crude odds ratio; ^f^the number of patients exposed to the prespecified CM during 13–16 weeks prior to the index date; ^g^the counts of concordant users were subtracted from total counts in case and control period 2 to give the numbers of discordant users for calculating the crude odds ratio; ^h^use of any prescription of the single CM or CM formula containing Asian ginseng, dong quai, danshen, ginger, licorice, or turmeric; ^i^adjusted for all comorbidities and comedications except cytoprotective agents; ^j^adjusted for all comorbidities and comedications; ^k^use of any prescription of the single Asian ginseng, dong quai, danshen, ginger, licorice, or turmeric; ^l^use of any prescription of the CM formula containing Asian ginseng, dong quai, danshen, ginger, licorice, or turmeric; ^m^use of any prescription of the single CM or CM formula containing the specified CM; ^n^adjusted for all other specified CMs, all comorbidities and comedications except turmeric, and cytoprotective agents; ^o^adjusted for all other specified CMs, all comorbidities, and comedications. ^*∗*^Statistically significant difference.
