# Supplementary material for: Uncertain Associations of Major Bleeding and Concurrent Use of Antiplatelet Agents and Chinese Medications: A Nested Case-Crossover Study
Source: Evid Based Complement Alternat Med. 2017 Aug 2;2017:9417186. doi: 10.1155/2017/9417186 (PMC5558644; doi:10.1155/2017/9417186)
Supplement: Supplementary file 1 — Appendix 1: The detailed use pattern of any of the prespecific Chinese medications during 2012-2013. Appendix 2: Results of sensitive analyses using the cohort with the same criteria and same case and control periods of 2010-2011 databases. [file 9417186.f1.doc]

**Appendix 1. The detailed use pattern of any of the prespecific Chinese medications in each study period during 2012-2013.**

| Control Period 1^b^ | Case Period ^a^ |
| --- | --- |
| Control Period 2^c^ | exposure days > 14 days  8 ＜ exposure days ≦ 14 days  exposure days ≦ 7 days  The number in each box was the patient identification (e.g., 1 to 62 in the case period). The small the number the close prescription date of specified CMs prior the hospitalization due to major bleedings.  * Patient had more than one exposure to CM with exceeded seven days intervals during each period. |

^a^ 1–4 weeks prior to the index date. ^b^ 6–9 weeks prior to the index date. ^c^ 13–16 weeks prior to the index date.

**Appendix 2. Results of sensitive analyses using the cohort with the same criteria and same case and control periods of 2010–2011 databases.**

**TABLE 1: The period prevalence and incidence of major bleeding among antiplatelet users.**

|  | Period prevalence | | Incidence | | |
| --- | --- | --- | --- | --- | --- |
|  | Patient number | %^a^ | Patient number | %^a^ | Per 1000  person-years^b^ |
| Ever encountered bleeding events | 1625 | 2.18% | 1327 | 1.78% | 15.9 |
| Gastrointestinal bleeding | 1182 | 1.58% | 980 | 1.31% | 11.7 |
| Urogenital bleeding | 157 | 0.21% | 121 | 0.16% | 1.4 |
| Intracranial bleeding | 127 | 0.17% | 108 | 0.14% | 1.3 |
| Nose and eye bleeding | 65 | 0.09% | 42 | 0.06% | 0.5 |
| Other bleedings | 109 | 0.15% | 87 | 0.12% | 1.0 |

^a^ The total number of patients included in the cohort was 74,682.

^b^ The total number of person-years was 83,644.

**Table 2 Comparisons of comorbidities and concomitant medications among antiplatelet users between case period and control periods during 2010-2011.**

|  | Case period^a^ | Control period 1^b^ | *p* value | Control period 2^c^ | *p* value ^d^ |
| --- | --- | --- | --- | --- | --- |
| **Comorbidity** |  |  |  |  |  |
| Hypertension | 496 (37.38%) | 404 (30.44%) | 0.0002* | 398 (29.99%) | <0.0001* |
| Coronary artery diseases | 319 (24.04%) | 283 (21.33%) | 0.0952 | 241 (18.16%) | 0.0002* |
| Diabetes | 204 (15.37%) | 190 (14.32%) | 0.4447 | 205 (15.45%) | 0.9571 |
| Renal failure | 124 (9.34%) | 106 (7.99%) | 0.2143 | 95 (7.16%) | 0.0408* |
| Heart failure | 100 (7.54%) | 83 (5.25%) | 0.1928 | 84 (6.33%) | 0.2215 |
| Cerebrovascular accident | 60 (4.52%) | 63 (4.75%) | 0.7818 | 54 (4.07%) | 0.5657 |
| Cancer | 59 (4.45%) | 52 (3.92%) | 0.4973 | 48 (3.62%) | 0.2777 |
| Liver disease | 55 (4.14%) | 38 (2.863%) | 0.0727 | 36 (2.71%) | 0.0427* |
| Obesity | 1 (0.08%) | 0 | 0.3172 | 0 | 0.3172 |
| **Concomitant medication** |  |  |  |  |  |
| Increasing risk of bleeding |  |  |  |  |  |
| NSAIDs | 1098 (82.74%) | 1010 (76.11%) | <0.0001* | 968 (72.95%) | <0.0001* |
| Statins | 357 (26.90%) | 348 (26.22%) | 0.6924 | 318 (23.96%) | 0.0821 |
| Glucocorticoids | 124 (9.34%) | 96 (7.23%) | 0.0487* | 86 (6.48%) | 0.0063* |
| SSRI | 55 (4.14%) | 55 (4.14%) | 1.0000 | 51 (3.84%) | 0.6917 |
| Warfarin | 38 (2.86%) | 34 (2.56%) | 0.6327 | 31 (2.34%) | 0.3932 |
| Prevent risk of bleeding |  |  |  |  |  |
| H2 blockers | 203 (15.30%) | 151 (11.38%) | 0.0030* | 134 (10.10%) | <0.0001* |
| PPI | 68 (5.12%) | 48 (3.62%) | 0.0576 | 36 (2.71%) | 0.0014* |
| Cytoprotective agents | 9 (0.68%) | 6 (0.45%) | 0.4373 | 4 (0.30%) | 0.1645 |

NSAID: Nonsteroidal anti-inflammatory drugs; SSRI: Selective serotonin re-uptake inhibitors; PPI: Proton-pump inhibitors.

^a^ 1–4 weeks prior to the index date.

^b^ 6–9 weeks prior to the index date.

^c^ 13–16 weeks prior to the index date.

^d^ Significance is reached when *P <0.05.

**TABLE 3: Potential risk of major bleeding associated with concurrent use of pre-specified Chinese medications with antiplatelet agents^a^**.

| CM^b^ | Number of users in different periods  (Case versus control period 1) | | | Exposure Odds Ratio  (95% Confidence Interval) | | Number of users in different periods  (case versus control period 2) | | Exposure Odds Ratio  (95% Confidence Interval) | |
| --- | --- | --- | --- | --- | --- | --- | --- | --- | --- |
|  | case period^c^ | control period 1^d^ | both periods 1^e^ | Crude | Adjusted | control period 2^f^ | both periods 2^g^ | Crude | Adjusted |
| Any of pre-specified CM^h^  Any of pre-specified single CM^k^  Any of pre-specified CM formula^l^  Licorice^m^  Ginger^m^  Asian ginseng^m^  Dong quai^m^  Danshen^m^  Turmeric^m^ | 86  22  85  83  67  51  49  12  1 | 72  15  69  67  55  39  44  9  0 | 51  11  49  47  34  25  27  5  0 | 1.67 (0.97-2.86)  2.75 (0.88-8.64)  1.80 (1.04-3.11)*  1.80 (1.04-3.11)  1.57 (0.91-2.72)  1.86 (0.97-3.56)  1.29 (0.69-2.44)  1.75 (0.51-6.0)  - | 1.61 (0.90-2.87)^i^  3.44 (1.02-11.64)*^i^  1.72 (0.96-3.08)^i^  1.66 (0.68-4.07)^n^  0.89 (0.38-2.06)^n^  1.30 (0.54-3.16)^n^  0.91 (0.39-2.15)^n^  1.51 (0.40-5.72)^n^  - | 65  14  61  60  47  36  36  7  1 | 34  5  33  32  24  17  19  2  0 | 1.68 (1.08-2.62)  1.89 (0.84-4.24)  1.86 (1.17-2.94)*  1.82 (1.15-2.89)*  1.87 (1.13-3.10)  1.79 (1..02-3.14)*  1.77 (0.97-3.20)  2.00 (0.68-5.86)  1.00 (0.06-15.99) | 1.53 (0.94-2.50)^j^  1.14 (0.48-2.73)^j^  1.80 (1.08-3.00)*^j^  1.15 (0.42-3.18)^o^  1.08 (0.40-2.91)^o^  1.24 (0.54-2.86)^o^  1.44 (0.63-3.30)^o^  1.14 (0.33-3.93)^o^  0.39 (0.02-8.91)^o^ |

CM: Chinese medication; OR: Odds ratio; CI: Confidence intervals.

^a^ The total number of participants included in case-crossover study was 1,327.

^b^ There were no patients prescribed with American ginseng, garlic, and Siberian ginseng during the case or control period.

^c^ The number of patients exposed to the pre-specified CMs during 1–4 weeks prior to the index date.

^d^ The number of patients exposed to the pre-specified CMs during 6–9 weeks prior to the index date.

^e^ The counts of concordant users were subtracted from total counts in case and control period 1 to give the numbers of discordant users for calculating the crude odds ratio.

^f^ The number of patients exposed to the pre-specified CM during 13–16 weeks prior to the index date.

^g^ The counts of concordant users were subtracted from total counts in case and control period 2 to give the numbers of discordant users for calculating the crude odds ratio.

^h^ Use of any prescription of the single CM or CM formula containing Asian ginseng, dong quai, danshen, ginger, licorice or turmeric.

^i^ Adjusted for all comorbidities and co-medications except cytoprotective agents.

^j^ Adjusted for all comorbidities and co-medications.

^k^ Use of any prescription of the single Asian ginseng, dong quai, danshen, ginger, licorice or turmeric.

^l^ Use of any prescription of the CM formula containing Asian ginseng, dong quai, danshen, ginger, licorice or turmeric.

^m^ Use of any prescription of the single CM or CM formula containing the specified CM.

^n^ Adjusted for all other specified CMs, all comorbidities and co-medications except turmeric, and cytoprotective agents.

^o^ Adjusted for all other specified CMs, all comorbidities and co-medications.

* Statistically significant difference.
